# Taxonomy of delays in the implementation of hospital computerized physician order entry and clinical decision support systems for prescribing: a longitudinal qualitative study

**DOI:** 10.1186/s12911-016-0263-x

**Published:** 2016-02-24

**Authors:** Hajar Mozaffar, Kathrin M. Cresswell, Lisa Lee, Robin Williams, Aziz Sheikh, On behalf of the NIHR ePrescribing Programme Team

**Affiliations:** Centre for Medical Informatics, Usher Institute of Population Health Sciences and Informatics, The University of Edinburgh, Doorway number 3, Teviot Place, Edinburgh, EH8 9AG UK; Institute for the Study of Science, Technology and Innovation, The University of Edinburgh, Doorway number 3, Teviot Place, Edinburgh, EH8 9AG UK

**Keywords:** Adoption, Delays, Health Information Technology, Implementation

## Abstract

**Background:**

Implementation delays are common in health information technology (HIT) projects. In this paper, we sought to explore the reasons for delays in implementing major hospital-based HIT, through studying computerized physician order entry (CPOE) and clinical decision support (CDS) systems for prescribing and to develop a provisional taxonomy of causes of implementation delays.

**Methods:**

We undertook a series of longitudinal, qualitative case studies to investigate the implementation and adoption of CPOE and CDS systems for prescribing in hospitals in the U.K. We used a combination of semi-structured interviews from six case study sites and two whole day expert roundtable discussions to collect data. Interviews were carried out with users, implementers and suppliers of CPOE/CDS systems. We used thematic analysis to examine the results, drawing on perspectives surrounding the biography of artefacts.

**Results:**

We identified 15 major factors contributing to delays in implementation of CPOE and CDS systems. These were then categorized in a two-by-two delay classification matrix: one axis distinguishing tactical versus unintended causes of delay, and the second axis illustrating internal i.e., (the adopting hospital) versus external (i.e., suppliers, other hospitals, policymakers) related causes.

**Conclusions:**

Our taxonomy of delays in HIT implementation should enable system developers, implementers and policymakers to better plan and manage future implementations. More detailed planning at the outset, considering long-term strategies, sustained user engagement, and phased implementation approaches appeared to reduce the risks of delays. It should however be noted that whilst some delays are likely to be preventable, other delays cannot be easily avoided and taking steps to minimize these may negatively affect the longer-term use of the system.

## Background

Policymakers in many countries consider health information technologies (HITs), such as computerized physician order entry (CPOE) and computer decision support (CDS) systems, to be critical attempts to achieve safer, higher quality, and more efficient health care [1–6]. With growing appreciation of the potential benefits of such systems, substantial investments are being made into HIT and this is translating into widespread adoption of these systems by hospitals [7]. However, as in other industries, implementations of IT are often delayed beyond their initially estimated delivery times [8–11]. In fact, evidence from a large number of studies suggests that more than half of IT projects fail to meet their initially estimated budget and/or timelines [12]. Delays in information system projects may increase the risk of not achieving organizational goals [13–15]. Ross and Vitale [16] describe an extreme case, in which an implementation overran timelines so much that no one wanted the system by the time it went live. In this case, interfacing a large number of disparate systems in the course of implementing an enterprise system led to unexpected integration testing times, which in turn contributed to the eventual non-acceptance of the system [16].

Delays are particularly pervasive in HIT implementations [17]. Projects overrun their timelines as implementations are often planned by people who are detached from the reality of delivering care [18]. Implementation delays not only cause extra financial burdens, but they also can lead to low morale among NHS staff [19] and impair end-user acceptance and effective use of systems, which could eventually lead to ‘failure’ or removal of the system [20]. Also delays can cause frustration to all participating organizations, ranging from users to governments and funding bodies [21]. This will also call for extensive review of the programs and widespread public criticisms of the process [22]. However despite the prevalence of delays and their potential consequences, there are very few studies that explicitly examine the causes of these delays. Failure to understand these reasons results in the same issues and problems being repeated over and over again [20]. The few available studies, which are around wider enterprise information systems, report on the implementation success factors, in which meeting project timelines is seen as one of the indicators of success. Such studies mention appropriate planning, minimum changes to the system, adequate mix of developers (IT implementation team members) and non-IT staff (e.g., decision makers and end users), and appropriate training to play a role in completion of the projects on-time and on-budget [23–30]. While useful, these studies have three main shortcomings which will be addressed in the current paper. First, they tend to report the local and immediate issues that arise during a project implementation time and ignore the supply side [31]. Second, as the focus of these studies is not on the timeliness of the implementations, they fail to offer a comprehensive list of causes. Third, they have a wider enterprise system focus, which falls short of explaining the particularities of hospital-based HIT implementations.

With the increasing scale and complexity of IT investments in health care in many developed countries, it has become clear that delays are the norm. Understanding the types and causes of delays and their clinical, financial, and organizational implications is therefore crucial – and can help to ameliorate potential adverse consequences. Hence, the purpose of this paper is to explore the causes of hospital-based IT implementation delays and develop a taxonomy of delays. This needs a more nuanced view which examines the complex socio-technical aspects and relationships involved in the process [20]. In order to achieve this and to address the above mentioned issues, we report on our study of the implementation of CPOE and CDS prescribing systems in hospitals. This work is a part of a national evaluation of CPOE and CDS systems in the UK hospitals, commissioned by England’s National Institute for Health Research (NIHR). CPOE and CDS are amongst the systems which have attracted the most attention from scholars in the field of HIT [32, 33] as they are potentially very transformative and have complex sociotechnical consequences [34]. They, together with Electronic Health Records (EHRs), are known to be amongst the most complex HITs which are commonly expected to bring the biggest benefits to patients and healthcare providers [35, 36]. As a result, despite the increasing interest in their implementation in hospitals, they are facing a wide range of challenges to be implemented in timely manner [37]. They are therefore likely to offer some important potentially transferable lessons in relation to other complex potentially transformative commercial HIT interventions.

## Methods

We conducted a longitudinal, qualitative multi-site case study investigating the sociotechnical challenges leading to delays in CPOE and CDS system implementation [38, 39]. We considered any type of implementation time overruns (actual time taken to implement compared to planned project time) as a delay.

### Institutional review board approval and ethical considerations

We received guidance from the National Health Service (NHS) Health Research Authority National Research Ethics Service (NRES) Committee London on 6 August 2012 that the study did not require NHS Research Ethics Committee approval as the work was classified as a service evaluation. As per our standard practice for studies that fall outside of the remit of NHS NRES, we therefore obtained ethical approval from The University of Edinburgh’s Research Ethics Committee dated 18 February 2013. Participants gave their written informed consent to participate. Data were anonymized for analysis.

### Sampling and recruitment

We used purposeful sampling to select six study sites that were planning to implement or had recently implemented three different types of CPOE/CDS systems [40, 41]. Our focus was on packaged applications, rather than bespoke systems, due to their rapid current and expected future growth in the UK and international markets [22, 42–44]. The case studies were chosen with a criterion sampling strategy [45] based on two parameters. The first criterion was our ‘typology of systems’ such that we selected at least one system from each of the following categories: standalone system (also known as interoperable systems), module within an integrated system, and distributed functionality amongst several modules [42]. The second criterion was the maturity of system in the U.K. market – i.e., we chose systems which were new on the market as well as systems which had several implementations in progress or were already used in several hospitals. Table 1 shows a description of each case.

**Table 1:** Summary of the case study sites

Site	Site characteristics	System description	Delay
A	Urban, acute care, university hospital 330,000 patients per year	- CPOE/CDS is a standalone application - System is widely used by several English hospitals, further implementations in progress	Project delayed less than 6 month
B	Urban, acute care 3,000,000 patients per year	- CPOE/CDS as part of an integrated hospital information system - System has several implementations in progress and is live in several English hospitals	Project delayed less than 6 month
C	Urban, acute care, university hospital 750,000 patients per year	- CPOE/CDS as a standalone application, interfaces built to enable decision support and interoperability with the wider hospital information systems - System is widely used by several English hospitals	Project delayed less than 6 month
D	Urban, acute care, university hospital 3,500,000 patients per year	- CPOE/CDS as part of an integrated hospital information system - Part of a joint procurement program - System has several implementations in progress and is live in several English hospitals	Project delayed less than 6 month
E	Urban, acute care 550,000 patients per year	- CPOE/CDS as a standalone application - System is new to UK market (no prior implementations)	Rollout delayed almost three years
F	Urban, acute care, university hospital 540,000 patients per year	- CPOE/CDS as part of an integrated hospital information system - Part of a joint procurement program - System has several implementations in progress and is live in several English hospitals	Project delayed less than 6 month

These different parameters helped us to investigate different dimensions causing delays for systems with differing architectures. We then contacted the Director of Pharmacy or Lead Pharmacist for HIT and used purposive and snowball sampling to recruit a diverse range of stakeholders to be interviewed [41]. To address the potential risks of bias caused by snowballing, after our first contact with Director of Pharmacy or Lead Pharmacist for HIT, we used respondent-driven sampling to maximize the chances of recruiting a maximum variation sample [46] to include a range of different respondents (e.g., varying levels of seniority, different professions, and different viewpoints). Our sample consisted of implementation team members and users of the system – in particular, those users who were involved in implementation.

### Data collection

We used semi-structured interviews as the main method of data collection. We supplemented these data with data collected from expert roundtable discussions. This was done to take into account as many reasons for the delays as possible from the perspective of both adopters and suppliers of technology [47, 48]. We also collected relevant project documents, these include implementation risk logs, roll-out plans, and business cases. Combining different methods enabled us to triangulate the data sources to validate emerging findings [49].

We conducted 214 interviews to obtain data about implementation and adoption of CPOE and CDS systems with a total of 163 interviewees. Some interviews were conducted with two or three participants simultaneously. Interviews were conducted at up to three time points in each organization. In the three hospitals that implemented the system during our study (Sites B, C, and F) we conducted: one set of interviews prior to implementation, one set just after the roll-out, and one set approximately one year post-implementation. In the hospital with the very long delay (Site E), we conducted three rounds of data collection at approximately one year intervals. However, as the system did not go live in the duration of our study (due to the delays), all three rounds of data collection were in fact pre-implementation. In the two hospitals that already had embedded systems (Sites A and D), we conducted two sets of interviews with an approximate 18 months gap between the two rounds. Table 2 provides a summary of data collection from each site.

**Table 2:** Summary of the interviews

Site	Time 1	Time 2	Time 3
A	23 interviews with 24 interviewees (19 clinical staff and 5 implementation team)	9 interviews with 10 interviewees (5 clinical staff and 5 implementation team) 5 interviewed before	
B	24 interviews with 24 interviewees (17 clinical staff and 7 implementation team)	17 interviews with 17 interviewees (12 clinical staff and 5 implementation team) 8 interviewed before	
C	13 Interviews with 13 interviewees (7 clinical staff and 6 implementation team)	18 interviews with 18 interviewees (15 clinical staff and 3 implementation team) 8 interviewed before	20 interviews with 20 interviewees (17 clinical staff and 3 implementation team) 10 interviewed before
D	15 interviews with 15 interviewees (8 clinical staff and 7 implementation team)	11 interviews with 11 interviewees (7 clinical staff and 4 implementation team) 10 interviewed before	
E	19 interviews with 19 interviewees (19 clinical staff and 9 implementation teams)	7 interviews with 6 interviewees (3 clinical staff and 3 implementation team) 1 interviewed before	3 interviews with 3 interviewees (3 implementation team) 1 interviewed before
F	15 interviews with 18 interviewees (14 clinical staff and 4 implementation team)	12 interviews with 12 interviewees (9 clinical staff and 3 implementation teams) 10 interviewed before	8 interviews with 8 interviewees (6 clinical staff and 2 implementation team) 6 interviewed before

The interviews consisted of open-ended questions about the procurement, implementation process and challenges, and use of the system. The topic of project timing and delays emerged spontaneously during the discussions and was followed up as a theme. The interview guides were therefore tailored to the roles/organizations of individuals to explore the reasons and implications of time overruns. Each interview took between 15 min to two hours (the mean time was 29 min). Data were collected between December 2011 and August 2015.

We also conducted two full day expert round-table discussions. Both meetings were held in Birmingham, U.K., and they were moderated by members of our research team. The first meeting (known as the business case workshop), held in October 2012, involved 17 participants including policymakers, clinicians from different hospitals, representatives of suppliers, academics, and patients. In this meeting, participants discussed topics around conceptualization and planning of implementations, specification of system functionality and drafting a business cases. The second meeting (known as the supplier workshop) had nine participants, representing six key vendors operating in England. In this meeting, the supplier representatives discussed issues, challenges and opportunities arising from the early stages of planning and procurement of CPOE/CDS systems to their implementation, use and long-term optimizations.

Interview and roundtable meeting data were digitally audio-recorded with written consent from participants and transcribed verbatim by a professional transcriber. The researchers, also recorded field notes for each meeting and interview.

### Data analysis

We drew on the biography of artefact (BoA) perspective to capture views of both adopters and suppliers of CPOE/CDS products and to develop longitudinal insights into how outcomes were shaped by prior sets of decisions [31, 47, 48].

We used NVivo 10 to analyze the data. A combination of thematic inductive and deductive analysis was used to examine the collected data and identify the main themes. We drew on a coding framework, based on the BoA, surrounding the technology lifecycle to build our themes. BoA informed our work in several ways [31, 47]: 1) to move beyond a single timeframe and evaluate the situation from initiation to use (by conducting a longitudinal study that examined the immediate as well as long-term consequences); 2) to move beyond a ‘localist’ perspective (by including the perspectives of adopters, suppliers, and policymakers and forming both the external and internal factors as explained in the results section); and 3) to examine both technical and non-technical aspects of the system. The BoA perspective allowed us to better understand and classify the causes and drivers of project overrun.

Considering the above mentioned aspects, initially the trained qualitative lead researcher for each case study (HM, KC, LL) analyzed each case separately to form the themes. We then conducted cross-case comparisons during analysis meetings held amongst all authors. In order to do this, we initially read each transcript and then examined it in relation to other transcripts. This allowed us to search across data sets and identify patterns [50].

## Results

The length of delays varied substantially in our cases ranging from one month to several years (more than three years). However, in all cases we found a combination of causes leading to time overruns. We identified 15 main interrelated reasons (shown in Table 3) leading to implementation delays.

**Table 3:** Taxonomy of Implementation Delays

	Unintended	Tactical
Internal	- Unrealistic or unclear business cases - Lack of detailed planning - Limited knowledge of CPOE/CDS - Human resource capacity - Poor user training -Weakness of broader information infrastructure	- Development of other applications - Customization and modification of applications - Interfacing with other applications
External	- Implementation in other adopting organizations - Limited supplier capacity - Safety issues	- Change of supplier organizational structure - Supplier strategies in responding to user needs - External contractual obligations

Drawing on our analytical framework, closer examination of this long list of delays revealed two main categories: delays caused by issues arising within the adopting hospital and delays originating from outside the adopting hospital (including other adopters and technology suppliers). As we analyzed the results further, another cross-cutting distinction emerged: unintended delays versus tactical delays. Unintended delays refer to unplanned and unexpected issues arising during the implementation which prevented the system progressing towards ‘go-live’, whereas tactical delays appeared to result from strategic longer-term planning decisions, often geared to achieving smoother or more dependable implementation and use. Hence, whilst unintended delays caused hindrance to going live, tactical delays were more deliberate strategies which addressed the long-term use of the system. Although we have categorized the delays into this taxonomy, we acknowledge that some types of delays which are attributed to one source may need to be resolved by another – for example, the adopting hospital may have had concerns about safety, which needed new developments by supplier to overcome the safety issue. In such cases, we classified the cause in the category of factors that adopter perceived to have originally caused the delay.

Table 3 presents a two dimensional model of the taxonomy of delays showing the classification of causes in relation to internal versus external factors and unintended versus tactical delays.

### Unintended internal delays

The first set of delays were caused by matters arising within the adopting organization.

### Unrealistic or unclear business cases

First of all, unrealistic or unclear business cases were said to be the primary cause of slipping on the originally planned implementation timeline. Due to the complexity of the CPOE and CDS implementation, it was critical for the project team to initially develop a precise business case which incorporated visions of goals and organizational improvements, and justification for the system. Such a business case enabled both suppliers and adopters to gain an overall understanding of their own as well as each other’s needs and offerings. However, due to shortage of capacity and resources in hospitals, business cases were often developed too quickly and procurements were made without adequate understanding of the problems needing to be addressed, which in turn led to unrealistic timeframes being specified.

*…your outline business case should [be] clear… [you need to have] put your business case together, justified the finances, justified the patient safety quality and other issues, got an outline of what you plan to do, issues around the pre-implementation work you don’t need it all process mapped but you need a clear understanding of what’s required, resources, skill mix on their side, not so much on the supplier side but on their side in order to go ahead, and so that sort of readiness-check type approach and an outline business case that should be clear that it should go to market, it shouldn’t go to market until they’ve done all that. (Supplier Workshop, Participant 7)*

This forced suppliers to enter into contracts without sufficient prior planning, understanding and agreement from both sides.

*…there’s a klaxon going off in my mind because if they’re (hospitals) not prepared to share the outline business case with you… There’s a problem with the contracting process again here, going back to contracting because I think we’re, to ask the question from a supplier point of view you’re boxed into a corner to sign up to an unrealistic project plan… (Supplier Workshop, Participant 8)*

The lack of clarity in developing business cases further led to diverse translations of the visions by different stakeholders, ranging from better management of data and processes to achieving a patient-centric health service. Such views resulted in lack of ability to align system configuration with long-term goals.

*… so the question is not so much what’s there now… but it’s also about what’s there now and how it’s going to align over a longer period of time with a whole load of other changes coming through, so does the procurement process take that into account, the health service procurement process or is it just a single one off purchase that they do with it? (Business Case Workshop, Participant 10)*

### Lack of detailed planning

A high level business case on its own was seen as not allowing detailed execution of steps on the ground. In several cases, we found that neither vendors nor adopting hospitals drew up detailed project plans. This led to inaccurate implementation time calculations and impractical implementation plans. Despite the necessity to have clear and reasonable milestones, some adopting hospitals were not rigorous in defining or enforcing manageable timelines at the early stages of the project.

*…the high level planning that went on to support the business case […] was at a very high level, it was blocks of time and it was an estimate based on relatively little information… So the first job I had to do when I got here was to take the high level plan which was effectively dots on an Excel worksheet that were months and break that down to a 500 line Microsoft Project plan with individual tasks and individual responsibilities and durations, start and finish dates and everything else… So once you start to look at the detail for the individual tasks you get a project plan that extends quite a bit further than you first anticipated… (Site E, Senior Project Manager)*

Also, in some cases, plans were highly aspirational and did not present the reality of everyday care provision. This lack of alignment between “must-haves” and peripheral functions (or “want-haves”) led to higher costs of implementations and possibly contributed to suppliers failing to meet the original requirements.

*It’s that incremental process isn’t it? There are certain [features] which are very much aspirational, there are certain ones which are must haves and there are certain ones that are a bridge between those two ends of the spectrum and you need to be able to migrate from the must haves into the aspirational wants if they’re still relevant… So if you allow people to specify all of their aspirational wants, their wish list then any supplier will eventually turn round and say yeah we can do all of that, this is how much it will cost you, at which point most NHS organizations will turn round and go we can’t have that one… (Business Case Workshop, Participant 2)*

Some suppliers had begun to address these issues by suggesting stepwise implementations: i.e., implementing and going-live with the basic functionalities first and then implementing more complex functionalities (e.g., decision support). They suggested that this would enable hospitals to gain an understanding of what was to be developed, allow them to better plan for their needs and as a result have better project time and resource management.

*I think it’s much better from my experience to go live with something which is relatively simple, potentially replicates paper so you roll out this advance functionality be it drug checking, adverse drug reaction forms that kind of stuff after the initial adoption stage has pitted out I guess. (Supplier Workshop, Participant 5)*

Further to the above, the lack of detailed planning added to the delays already being experienced. This meant that delays became longer as a result of the yearly patterns of change/patient fluctuation that occurred in the case sites. Winter pressures meant that when a project was delayed by a few months from summer it was unlikely that the site would be able to start its roll-out until around February (hospitals chose to delay roll-out until after the winter busy times as the pressures could lead to complications at go-live), so a delay of two to three months turned into one closer to six months.

### Limited knowledge of CPOE/CDS

A lack of understanding and experience around the system being acquired and its implementation challenges, coupled with reliance upon high level business cases without detailed implementation plans also led to underestimation of resources in hospitals.

*I think the staffing needs that we started with are very different […] I think we underestimated how much support we’d need in terms of data analysis and informatics, that’s probably something we didn’t think about very much at the beginning. (Business Case Workshop, Participant 7)*

As a result, as implementations proceeded, there was often a need to increase staffing to deliver the project.

*So it’s just learning for us as well of how to roll out and what support [is needed]… Because sometimes you plan for things, and then you just suddenly realize actually we need to do this a bit more. (Site D, Pharmacist)*

Hospitals relative unfamiliarity with many of the CPOE and CDS systems in the UK market added to the issues caused by complexity, leading to limited understanding by adopting organization about the nature of the COPE and CDS products. This lack of awareness about project scale, complexity of implementation process, and systems was identified as another major reason for lack of detailed planning.

*…until you know what the product looks like and how it works and how it functions and how long it takes to deploy it you can’t do detailed planning and obviously, and this is not a criticism but the people that procured the product very few of them knew anything about how to deploy an electronic prescribing product… (Site E, Senior Project Manager)*

### Shortage of required human resources

Implementations also overran due to issues with human resources, which were particularly intractable as projects spanned many departments and professional groups across the organization. These complex projects thus required an implementation team that spanned the organization and continued to support the implementation over an extended period. However, some hospitals lacked the local expertise needed in the implementation team. They failed to recruit people on time or lost project employees during the course of the implementation.

*… the reason we slipped from our February go live initially, we wouldn’t have done it then anyway, to April and then May is we didn’t have our full team in place, we couldn’t recruit them. (Site C, Project Manager)*

These teams sometimes had difficulty in engaging the necessary stakeholder to execute their plans. On the one hand, certain professional groups were not involved/invited to become part of the implementation team or when they did, their input was seemingly ignored; on the other hand, there was a feeling among the implementation teams that there were many individuals who chose not to get involved (i.e., they deprioritized the CPOE/CDS implementation).

*Nursing as a whole has had a very small voice and one of the main reasons behind that and one of the significant issues that [Site F] has as an organization is that there’s a 50 % vacancy rate, so ultimately it’s very difficult to try and engage a workforce that’s very transient in its population and within its workforce… (Site F, Lead Nurse for HIT)*

The shortage of human resources seemed to arise because of two related underpinning factors. First of all, the particular employment practices in the health sector acted as a disincentive, discouraging individuals from taking up short-term contracts. It also dissuaded health professionals to use their newly gained expertise to change professional rout and become HIT consultants. This meant their expertise was barely transferred for implementation of HIT project in other health organizations.

*It would be quite common [in other sectors] to have independent consultants to advise a procurement in the private sector… We did have two people […] who’d gone from being implementation experts to being supply side players but is there a class of people who’ve implemented [CPOE/CDS] systems who then serve as [CPOE/CDS] consultants? If not why not, is it because you can’t pay for them? (Business Case Workshop, Participant 10)*

Secondly, funding issues made it difficult for hospitals to recruit the required staff for long-term.

*… NHS England is giving money over for a finite amount of time, there’s no money in there for roll out and the [hospitals] have got to think about this going forward because that project team that’s there for that implementation is going to go and unless you get to that point where the organization is mature enough to take that on board and move that forward and has the money to release those people, and we were talking conservatively at what £300,000 for staff going forward. So it’s not just about the IT structure and it’s not just about the project phase… it’s a long road. (Supplier Workshop, Participant 1)*

### Poor user training

We also found that in many sites, delays were caused by a lack of appropriate user training.

*But we know we have to do that quite carefully and with the appropriate training and support and systems in place otherwise we could be introducing significant risk to the patients. (Site C, Clinical Effectiveness and Medicines Manager)*

Simplistic assumptions of the extent and the type of training required for each profession and the time required by each individual to be released from daily activities led to new challenges and extended timelines. As a result, further staffing was required to deal with temporary absence of staff from their daily duties to attend training classes.

*I mean we’ve had some sort of difficulties with training only in as much as people being released to attend classroom training because for nurses it’s a whole day and for doctors it’s half a day. (Site D, Clinical Change Lead)*

We also observed problems with user engagement when trainers were not familiar with healthcare practices and settings.

*You do classroom based training and people don’t turn up that’s the problem. I have anecdotes here and tales that when you’ve got some senior clinicians being trained by somebody who’s not from a clinical background they’re switching off… (Site D, HIT Manager)*

### Weaknesses of the broader information infrastructure

Implementations were also delayed due to lack of appropriate infrastructures in hospitals.

*… Clearly you can’t go live with such a thing without having the appropriate infrastructure in place which was a major reason for the delays at the beginning. (Site D, Consultant)*

In many cases, the CPOE/CDS system relied upon the same HIT infrastructure that was used for other applications, and installed through organization-wide planning processes. As a result, planning of HIT capacity (hardware and networks) for CPOE/CDS became dependent on other HIT projects, which led to timelines being extended.

*… probably took three months for us to do our work and then maybe another month, month and a half to get machines ready because we identified that if we were going to go down this whole Terminal Server 2008 route machines had to be upgraded to the latest service pack and there was a few other little fixes that had to be done. Like if you’ve got Word open but you haven’t got it in full screen mode you can click it and drag it round your screen and what we found was if the machine weren’t service pack if they had something else if you dragged it would make like a drag mark on the screen and it would leave trace marks everywhere so we had to do like this prep work, again it was to make the end user experience better. (Site A, Implementation Team)*

### Tactical internal delays

We found that the second type of delays was due to tactical decisions taken by the adopting organization during the implementation period. We refer to these as tactical delays because they arose as a result of strategies adopted to enhance longer-term adoption and optimized use of the system. So whilst there was the choice of preventing delays caused by these reasons, organizations decided to take the risk and opt for a longer-term benefit.

### Development of other applications

Our findings show that implementations could be delayed as CPOE/CDS became dependent on the implementation of other applications. Such decisions were taken particularly with non-integrated systems. Here, decisions to delay the go-live were made by the adopter organization to achieve a broader goal (e.g., smoother flow of information between different systems).

*The biggest delay and the most significant one was obviously […] dependency on the delivery of the EDS [electronic discharge summary] software […]. If you look at this, just to illustrate this was an updated version of the plan but the software is going to be delivered here and we’ve got future stake workshops scheduled at the time the software is delivered. That can’t happen, we have to get the software, create the future stake process maps and then hold the workshops so this date is likely to go back and this date is therefore likely to move as well. (Site E, Senior Project Manager)*

In this case, usually third-party “bolt-on” software was required to extend the use of CPOE/CDS system.

### Customization and modification of applications

Customization was also responsible for delays. This type of delay was usually a result of: a) the CPOE/CDS system needing an element to run or Anglicization was needed to fit UK specific needs (e.g., the need for a particular UK specific requirements on ‘To Take-home Orders); or b) opportunities were identified to enhance the use of CPOE/CDS systems by modifying the built-in functionalities. In the first case, dependency on a particular element could be a necessity for the organization to perform (so less of a tactical choice but more of an imperative), whereas in the second case customization was a choice rather than a necessity (thus more of a tactical decision).

*I think there are probably quite a lot of problems with [system name]. In the first place, it came as quite an American product and it’s taken quite a long time to Anglicize it and make it more suitable for the UK market … there are quite a lot of NHS specific things which are not built. (Site D, Information and Communication Technology Manager)*

While this was classified as an internal issue (particularly when customizations were due to system enhancement), actions from suppliers were required to respond to such needs. Hence delays could occur due to limited supplier capacity or due to suppliers’ strategies in responding to user needs (discussed below).

### Interfacing with other applications

The need to develop interfaces between various applications was also an important cause of delays. We observed that, although the systems could go-live, their implementations were delayed to achieve the longer-term advantages of transferring data from CPOE/CDS to other existing systems.

*In theory, and I gather from [name of project team member] certainly last week it still wasn’t fully in place within the [system name – standalone] system but in theory they’ll be able to just pull it out from the [system name] system onto the discharge summary but she was saying two weeks ago that it wasn’t, that particular bit wasn’t in place yet but they were working on it. We’ve got a meeting with them tomorrow so I might find out more then, but in theory it should make [hospital medical records] writing easier because they can just pull it straight off the [CPOE/CDS] system but that’s not ready to roll yet, she’s concerned that could be one of the delays that would stop us from starting next week. (Site C, Senior Sister)*

Such interfaces, if designed well, could also be beneficial for the users of the system. However, there was also the possibility of several designs being tested to achieve the best result, which in turn could delay the implementation even further.

*That install was different in a number of different ways. We originally negotiated the contract so that [system 1 name] would be accessed using [system 2 name]… but it was the wrong decision to make, one, PAS [patient administration system] are not something that doctors use very much, doctors tend not to use PAS systems, two, is any changes to the user interface would require us to negotiate with [supplier of system 2] to get changes. We wanted to have a single point of access to all our clinical systems, clinical portal so we changed it so that we wish to invoke [system 1 name] which is the [supplier of system 1] product from within our clinical portal rather than from within {system 1]… But it does change what was deployed because they had to install additional software. We have changed where we wish to capture things like allergies, and height and weight and all of these were changes to the scope. So basically we’ve restarted the project as I mentioned with effect from last week… (Site E, Senior Project Manager)*

Although such challenges were mainly observed in standalone systems, integrated systems presented similar issues, particularly when two-way interfacing between hospitals and other health and care organizations was required. Furthermore, the challenges became even more when customization of integrated systems was needed. This was because of the higher complexity of integrated systems resulting from tightly coupled dependencies that existed between different modules and their underlying databases.

In general all types of application adaptations – extension, customization, or interfacing – caused long delays if they were not planned for in the early stages. While extensions and interfacing were more evident in the case of standalone applications, customizations caused longer delays in integrated applications due to their higher complexity.

### Unintended external delays

While the above mentioned delays resulted mainly from internal issues or in-house strategies, we found that there were also unanticipated delays caused by the external environment.

### Implementation in other adopting organizations

Procurement of solutions by other hospitals created a new source of delay, as implementations were paused awaiting resolution of implementation problems in other adopting organizations. Due to parallel implementations of one product in several hospitals, implementation in one (or a few) hospital(s) were delayed/paused as the supplier was implementing its product in other hospitals, creating a dependency between different organizations.

*One of the biggest dependencies for us is the readiness of the software and that is being driven by [another hospital name]… there is the critical dependency on delivery to [other hospital name] but leads to our project very directly so clearly where [other hospital name] was delayed that potentially causes delay to our work … They delayed their go live… we have a dependency on the same release of software. (Site E, Senior Project Manager)*

This was a major issue especially in CPOE/CDS products, which were new on the UK market due to differences between the systems functionalities and the needs of UK hospitals. As a result, suppliers had to go through complex cycles of market analysis, development and implementation at once. Also, joint procurements [51] led to further delays as a large number of adaptations in initial implementations were taking place all at the same time and suppliers had to deal with various requirements at once.

### Limited supplier capacity

As mentioned earlier (under the customization and modification of applications section), CPOE/CDS is still in its infancy in the UK, and systems are in the process of being anglicized [37]. However, we observed that limited supplier capacity resulted in delays of system implementation. This was a particularly important issue for overseas suppliers as they had a limited number of employees present in the UK. Thus, most of the development work had to be sent back to the countries of origin. This in turn, caused complications in capturing the needs of U.K. settings.

*[We were] the first to go live with meds management in [system name] in the UK fully. Therefore there was no front runner that you could say well yeah you’ve already built this for this [hospital…] so there was a limited resource in the [supplier name] world to assist the [hospital] in developing and deploying that product and that does cause delay definitely. (Site F, Interim director for HIT)*

Suppliers’ limited capacity led to lengthy responses to users’ requests. In some cases, adopting hospitals had to wait for months for a new release of the product to be ready before going-live. In other cases, hospitals went live with an older version in the hope that they would eventually upgrade to a release in which their requests were incorporated.

*So we have postponed by three months the going live on the first two wards in essence because the supplier hasn’t been able to have the product ready for use so you could say we’re a bit frustrated by that… And there are things like […] you can get log outs, so you could get access to certain patients blocked if certain events happen and that’s quite tricky and is taking time to get resolved. Their new version of software that is going to come will make that a much smoother process but that isn’t yet available, it should be available before we rollout further. (Site C, Clinical Effectiveness and Medicines Manager)*

### Safety issues

Lack of availability of system functionalities sometimes led to concerns about potential safety risks. In such cases, the adopting hospital made several efforts to delay the go-live date until a solution was achieved.

*There’s only one thing […] is that there is a facility to do a free format prescription. Say for example Parkinson’s medication and you need to give it at different times of the day, specific times of the day that don’t fit in with the drug rounds, or sort of a Methotrexate dose that is given weekly, you can actually select it weekly but say if the doctor did it in this free format and said Methotrexate 10 mg weekly and the system doesn’t understand that that’s weekly, it doesn’t attach it to a frequency so it then comes up every single day for admin but they’ve raised that with [supplier name] as a risk but without looking, obviously now we know that we would need to look out for it, we’ve got sort of an alert for any drugs that are prescribed free format but if you didn’t sort of look on that thing then you wouldn’t know and obviously the staff would change every day and they wouldn’t think oh this patient on Methotrexate yesterday because it would just come up what needed charting for that day, so there’s a real risk there… that’s not good enough particularly when a large number of issues that have been raised are around absolute patient safety. (Site A, Pharmacist)*

### Tactical external delays

The final type of delay was caused by changes of strategies and long-term plans by external entities (i.e., suppliers, third party organizations, and policymakers).

### Change of supplier organizational structure

Mergers and acquisitions often have a knock-on impact on companies external relationships to its customers [52]. Change of suppliers’ organizational structures which was a direct result of acquisitions, was another cause for delays in the implementation process.

*Unfortunately for us when I raised the RFC [request for change] a company called [company name] came in and took over [company name- original supplier] so there was a corporate takeover and that delayed the process. They’re an American company and they needed a PAS system for the UK market so they purchased [company name- original supplier] but obviously that resulted in a five to six week delay whilst we got the request for change result… (Site E, Senior Project Manager)*

Such changes could lead to contractual modifications or even strategic change of the application which in turn caused further delays.

*Lack of contract signing [by the new supplier] and unwillingness to continue to work at risk – this will likely end up in adding 2–3 months to the timeline… Issue between [supplier 1 name] and [supplier 2 name] contracts preventing deployment of software on existing hardware… (Site E, Project Document)*

### Supplier strategies in responding to user needs

Another type of tactical delay caused by external entities was caused by suppliers’ strategies in responding to user needs. In this respect, suppliers had “sorting and sifting” strategies in order to prioritize and plan for their future developments.

*…if we need something to happen we have to log it with a user group who then have to agree it’s something we want to take forward which then goes to the vendor, they decide whether they can be bothered to do it, it then gets on their work plan and maybe three years later we get our change… (Site A, Pharmacist)*

Suppliers prioritized the needs (including functional needs and resource needs) and even the customers, based on internal strategies which in turn led to late response to some of users’ requests.

*Anything which is dependent on [supplier name] takes months which is not ideal and we’re also limited by when they can put the changes in so it can be quite difficult. (Site F, Consultant)*

There were also delays caused by contractual issues enforced by the supplier. Suppliers sometimes introduced ‘freeze’ periods in the contracts which meant that no change requests could be put in for a certain period of time.

### External contractual obligations

Two case study hospitals were part of the NPfIT geographical clusters (North West/West Midlands, North East, East of England/East Midlands, London, Southern) programme [51]. This meant that a single contract with a local service provider (LSP) was signed for a joint development of information infrastructure, and as a result the supplier of CPOE/CDS was subcontracted by the LSP. In these cases, the National Programme created delays because of the structure of the contracts which meant that change requests could not go directly to the supplier and had to instead go first through the LSP. This arrangement added bureaucratic layers and thus slowed down the pace of implementation in these two sites. The contract also defined staffing levels and resource management strategies, which led to further implementation delays.

*[The contract] constrains or limits what the companies can respond to because they’re constrained by a fairly large contract and constraints around that so it defines both the staffing, the product and the services that you can get so you get a standard approach rather than a customized approach if you went directly. (Site F, Change Manager)*

### An overall picture of the taxonomy

The taxonomy of delays highlighted in this paper is developed based on the most frequent causes observed across the sites. Overall, we found that all types of unintended internal delays were evident in all six sites. Also, apart from development of other applications (which was a delay cause in Sites A, D, and E), all the other types of tactical internal delays occurred in all the sites. In contrast to internal delays, the external delays varied across sites. Implementation in other adopting organizations, limited supplier capacity, and change of supplier organizational structure caused delays only in Sites A and E. Supplier strategies in responding to user needs led to delays in Sites A, C and E. Finally, external contractual obligations and safety issues were seen as a delay cause in all the sites.

## Discussion

Delays in the implementation of HIT systems are extremely common, but poorly understood. To understand and classify these delays, we have sought to develop a taxonomy of delays in relation to CPOE and CDS systems that go beyond a single organization and capture longer-term dynamics between different stakeholders. Our taxonomy has the potential to help clarify important aspects of implementation and facilitate ways to prevent or mitigate the risk of such delays in future implementations. Our analysis revealed that delays may occur due to internal and external factors surrounding the adopting hospital. We found that time overruns may result from planned strategies as well as unplanned events. This work also shows that implementation delays are influenced by a number of risk factors including lack of knowledge and prior experience, poor project planning, fading user involvement, limitation of resources, dependencies due to standardization of systems (i.e., one supplier offering one product to a number of different users), immaturity of products and market, and changing structures and requirements.

### Strengths and limitations

Drawing on the BoA framework, which suggests studying organization and technology in tandem over several timeframe and locales, enabled us to reach an understanding of the broad range of delay causes gathered from views of suppliers, implementers, policymakers, and adopters over an extended timeframe. We collected data from six case studies with varying implementation strategies and different delay durations. We also sought to capture the range of offerings in the UK market encompassing both UK and non-UK systems and standalone and integrated solutions to capture the full range of delay types and causes. This study, which has built on our earlier findings on successful implementation and adoption of HIT systems [53], has been the first to report on issues within and beyond hospitals that lead to an inability to roll-out a complex HIT system in line with originally planned timelines. Although our findings are built around data collected from CPOE/CDS systems, our results and recommendations are likely to have implications for a range of other hospital information technologies such as electronic health records, specialized chemotherapy applications, and laboratory systems.

An important limitation of this study is that we have reported on the causes of delays in a yet growing CPOE/CDS market which means that as the systems become more mature new impinging factors may unfold. Another limitation was that it is difficult to define delays as they may occur at different points in a projects lifecycle [54] (e.g., delays in pilot wards versus delays in roll-out in all other wards). So we used an inclusive approach and captured any time overrun for the major projects milestones. Further studies are needed to validate the generalizability of the taxonomy for other commercial off-the-shelf HIT systems.

In planned follow-on work, we plan to extend this work on causes of delays to other HIT systems and also seek to identify approaches to minimize the risks and/or mitigate the adverse consequences of HIT implementation delays.

### Interpretation of findings in the context of the wider literature

In other industries, it has previously been noted that a lack of understanding of products and the market adversely influences performance [55] and execution [56] of a project which can contribute to long implementation delays. Hence knowledge transfer mechanisms must be established to lessen uncertainties that cause inaccuracies in time estimation which is one source of delay [57]. Moreover, simplistic assumptions in defining business cases and glossing over the complexities, without developing tailor-made objectives and clear requirements, results in inaccurate time assessments. So in the early stages of project procurement and planning hospital characteristics and its long-term strategies must be clearly defined [58]. In a yet immature and growing CPOE/CDS market [42] where understanding of the technology and knowledge about products on the market are limited, extreme efforts are required to align hospital processes and those of the vendor offerings. Mitchell [57] suggests that intra-organizational coordination is required to achieve alignment through knowledge integration and joint planning by involvement of suppliers and adopters.

The combination of uncertainties about products, adopters and demands in this immature market has led to a situation called ‘the waiting game’ [59]. On the one hand, for business cases and project plans to be more precise, hospitals need to refer to earlier implementation experiences and the availability of CPOE/CDS packages. On the other hand, products are not fully developed in a yet uncertain market. So there is a dilemma between going with existing systems and knowledge, and delaying the decision while the prospects of the innovation become clearer over time. As studies show, the CPOE/CDS market is still in its infancy [37] and strategies such as joint procurement of premature products bring associated costs [51]. As a result of premature procurements, further dilemmas occur around customization and interface requests and even around meeting safety risks. So there is a trade-off between early implementation versus waiting till requirements are met. Hence an ongoing coordination between emerging technologies and market are needed in order to overcome the waiting games which includes phased introduction of a novel technology till products stabilize and implementation experience increases [59].

Our study shows that delays arise in implementing and adopting both standalone and integrated solutions. Although some delays are apparent in both cases (such as shortage of human resources and user training issues), many other delays differ in character and frequency depending on the type of system. For instance interfacing issues generated long delays in implementation of standalone applications, in particular because interfaces were required between the CPOE/CDS system and many other existing applications in the hospital. In integrated applications, however, delays caused by interfacing were less frequent. Instead due to their complexity, customization and modification of the applications caused longer delays. Also project planning and staff engagement challenges are likely to become more pronounced in integrated applications, because more extensive infrastructures are likely to impinge on an increasingly wide range of functional departments and occupational groups. This illustrates an important trade-off between discrete systems and more complex integrated applications (Cresswell K, Mozaffar H, Lee L, Williams R, Sheikh A. Integration and interfacing of hospital health information technologies: a national qualitative study of hospital electronic Prescribing systems Submitted to BMJ Quality and Safety).

### Implications for policy, practice and research

Policymakers and practitioners have focused on procurement and implementation of HIT in hospitals. In the light of this evolving technology landscape and drawing on our research and wider international studies, we suggest that simplistic and unrealistic assumptions at the early stages of project are unhelpful in making decisions for procurement and planning of HIT projects. Adequate time and effort must be spent at early stages of project to capture the needs of short- and long-term users, system benefits and implementation strategies. We have obtained important insights about the causes of delays in CPOE and CDS implementations. As we have shown the type of system (i.e., integrated or standalone), maturity in the market, procurement strategy (i.e., joint versus single procurement), and implementation strategy lead to different risks (as well as benefits). To minimize the negative consequences of these risks, long-term perspectives are needed prior to procurement as well as in the course of implementation of these systems. Finally, we suggest that, as in this study we have investigated commercial systems that are now also being deployed in many hospitals throughout the world, the considerable complexity of these CPOE/CDS systems, which should therefore offer considerable insights into comparably complex systems such as EHRs, and the fact that our work was undertaken over a number of case study sites which enabled us to understand the reasons for the delays encountered, the findings of this study may be applicable to other commercial off-the-shelf HIT implementations and if so may help to minimize the frequency and consequences of project delays.

## Conclusions

To our knowledge, this is the first study to explicitly identify and classify the specific causes of delays and interruptions in implementation of CPOE/CDS systems. Our work also indicates that, whilst there are strategies to mitigate some project delays (such as detailed planning, acquiring better knowledge of the market and systems, and stepwise implementation strategies), many other delays are unavoidable. In particular, the immaturity of the CPOE/CDS products in the market adds to the complexity of estimating the time required to implement and of sticking to the plans. Finally, we highlight that some delays are deliberately adopted in the course of efforts to benefit the smooth implementation and longer-term usability and performance of the system. Long-term planning, starting prior to the procurement of systems and continuing as the system is implemented, is the key to minimize the risk of these delays. Whether to delay the go-live date and accept the consequences, or to roll-out the system and disregard the possible longer-term impacts is a complex decision.
